# The influence of social class and institutional relationships on the experiences of vaccine-hesitant mothers: a qualitative study

**DOI:** 10.1186/s12889-022-14420-1

**Published:** 2022-12-09

**Authors:** Lindsay W. Glassman, Julia E. Szymczak

**Affiliations:** 1 Mathematica, Princeton, New Jersey USA; 2 Department of Biostatistics, Epidemiology and Informatics, Perelman School of Medicine, University of Pennsylvania, 423 Guardian Drive, Philadelphia, PA 19104 USA

**Keywords:** Vaccine hesitancy, Vaccine refusal, Vaccine decision-making, Social class, Qualitative methods

## Abstract

**Background:**

Vaccine hesitancy is a growing problem in the United States. However, our understanding of the mechanisms by which socioeconomic status (SES) shapes the experience of vaccine hesitancy and decision-making is incomplete.

**Aim:**

The aim of this study was to understand how social class influences the experiences and perspectives of vaccine-hesitant mothers.

**Methods:**

We conducted semistructured interviews with middle- and working-class vaccine-hesitant mothers. Participants were identified through neighborhood parenthood groups in the Philadelphia area, as well as in-person and online groups whose members express concerns about vaccines. Interviews were audio recorded and inductively analyzed.

**Results:**

Interviews were conducted with 37 vaccine-hesitant mothers, who described their vaccine decision-making through the lens of interactions with three institutional stakeholders: 1) pediatric clinicians; 2) school administrators; and 3) emergency room staff. In discussing these interactions, middle- and working-class mothers invoked distinct levels of authority in relation to these institutions. Specifically, working-class mothers expressed concerns that medical or school professionals could act as reporters for state intervention, including Child Protective Services, while middle-class mothers did not. These interactions highlighted the ways middle- and working-class mothers in our study felt differently empowered and constrained in their vaccine choices, and ultimately influenced their perceptions of available actions.

**Conclusions:**

Our findings indicate that experiences of vaccine hesitancy may be influenced by mothers’ social class via their relationships to institutional authorities. These findings have implications for how clinicians communicate with parents from different social backgrounds to best build trust and facilitate vaccine uptake.

**Supplementary Information:**

The online version contains supplementary material available at 10.1186/s12889-022-14420-1.

## Introduction

Vaccine hesitancy is a growing problem in the United States. While vaccination rates remain over 90% for primary childhood vaccines like those for measles, mumps, and rubella (MMR) and Hepatitis B (HepB), [1] parental refusals are on the rise. [2] In 2018 the median non-medical vaccine exemption rate among kindergartners was 2 percent, with state-level non-medical exemption rates as high as 7.5 percent. [3] Additionally, between 19 and 28 percent of parents report that they are vaccine hesitant, including those with sufficient concerns to selectively refuse vaccines or delay the recommended schedule. [4] Being under- or unvaccinated negatively impacts children’s health by leaving them vulnerable to vaccine-preventable diseases, and by increasing the spread of vaccine-preventable diseases to others who are unvaccinated. [5].

That socioeconomic status (SES) influences parents’ vaccine decision-making has been established, [6] but our understanding of the mechanisms by which it shapes parents’ perspectives and experiences of that decision-making process is incomplete. Despite similar levels of vaccine hesitancy across SES levels, [7, 8] parental behaviors differ. White, college educated parents earning over $70,000 per year are more likely than parents of color and those without a college degree to refuse vaccines [9, 10] and to claim vaccine exemptions for their children in school. [11, 12] Conversely, parents without a college degree, of color, and below the poverty line are more likely to have undervaccinated children [13], often related to access barriers such as cost, insurance status, and appointment logistics. [6, 14].

To further specify the mechanisms that account for the relationship between SES and vaccination uptake, research that examines the impact of social context on parents’ vaccine decision-making experience is needed. [15] Using the sociological concept of "social class" to examine parents’ vaccine decision-making experiences can identify novel factors that shape vaccine hesitancy. [16] The term “social class” describes groupings of individuals determined by a combination of social status measures, including income, education, and occupation, which create shared experiences and ways of relating to the world. [17, 18] Thus, while social class draws on similar metrics to SES, it encompasses an additional degree of analytical complexity that acknowledges the way social status shapes collective culture, power, and behavior. [15, 19, 20] While social class is not frequently used in research on parental vaccine hesitancy, it provides an analytic lens through which to understand the collective experience of parents as they make medical decisions for their children. In particular, the concept of social class draws attention to the role of social networks in circulating information about decisions and the formation of norms about what it means to be a parent, dynamics that have been shown to be important in relation to vaccine decision-making. [16, 21] The objective of this study was to understand how social class influences the experiences and perspectives of vaccine-hesitant mothers.

## Methods

We conducted semistructured interviews with middle- and working-class mothers in the greater Philadelphia metropolitan area to understand how they navigated vaccine decisions for their children. This manuscript reports on the analysis of data gathered as part of a dissertation study in sociology conducted between 2014 and 2019. [22] The purpose of the dissertation was to understand how mothers make medical decisions for their children, with a focus on why some refuse specific medical interventions while others do not. As part of that dissertation study, we interviewed 87 mothers about their medical decision-making for themselves and their children, and conducted over 500 h of ethnographic observations of religious and secular groups that promote alternative medical/healing practices. We focused our study on mothers because they are overwhelmingly the key decision-makers regarding children’s health. [23, 24] Based on these interviews and observations, we explored mothers’ voluntary refusal of a number of medical interventions, including vaccines. In this manuscript, we report on an analysis of a subset of interviews from this larger dissertation study (*n* = 37) with mothers who expressed vaccine hesitancy to better understand their experiences and beliefs surrounding immunization for their children.

### Study participants

For the dissertation study, we sought to sample mothers in the greater Philadelphia metropolitan area who had refused medical interventions for their children and those who had not. Respondents were recruited from the general public and identified through two in-person support groups for those interested in alternative medicine (in which some, but not all members were opposed to vaccines), as well as one non-denominational conservative Christian church in which congregants refused vaccines. Participants were also identified through three Philadelphia neighborhood parent listservs, as well as one Facebook group devoted to vaccine refusal, and two Facebook groups devoted to refusing conventional medicine in favor of alternative medicine. All recruitment was conducted by the lead author, then a doctoral candidate in sociology at the University of Pennsylvania. Approximately one third of the sample was snowballed from previous respondents. All potential respondents were told that the researcher aimed to understand how parents make health decisions for themselves and their families, with a focus on if and what conventional medicine parents have ever refused.

### Data collection

Semistructured interviews were conducted using a guide containing open-ended questions designed to elicit participant perceptions with minimal prompting from the researcher. [25] The interview guide was created based on a review of existing literature as well as emergent themes identified during the first three months of ethnographic observations.[26] Questions were designed to elicit a narrative of mothers’ medical decision-making in the context of their social and family backgrounds, and focusing on mothers’ interactions with health information resources, such as physicians, alternative medical practitioners, other parents, and the internet (see Additional File 1 for the interview guide). We gathered data on self-reported race, age, occupation, and number of children. In order to maintain rapport with the interview respondent we did not ask for self-reported income, data whose exclusion we also justified since they are subject to a wide range of error properties. [27] Respondents were given the choice between an in-person or telephone interview. The lead researcher, a female sociologist with experience interviewing parents about their children's health, conducted all the interviews. Participants provided oral consent following a verbal presentation of the informed consent form, and retained a copy of the form for their records. All participants took part in a single interview, which was audio recorded with participants’ permission and transcribed verbatim. The lead researcher wrote field notes immediately following each data collection session, [28] and memos of emerging themes from each interview. [29] Memos were used to determine sample size adequacy. We ceased data collection once thematic saturation of each major interview question domain was achieved. [30] This study was approved by the Institutional Review Board at the University of Pennsylvania (Protocol #826,314).

### Data analysis

Transcripts were uploaded to Atlas.ti qualitative data analysis software for management and coding. [31] We used a combination of analytic open and focused coding strategies to analyze the interview transcripts. We began data analysis by creating a codebook that contained codes derived from the major domains in the interview guide, including health beliefs, clinical interactions, and decision-making processes around medical choices (including, but not limited to, vaccination). The codebook was revised to allow for the inclusion of new codes that emerged as salient from the transcripts. [32] All analysis was performed by the lead researcher, who wrote analytic memos while coding to explore emerging themes and identify supporting and falsifying data. [33] Participant demographic information and degree of refusal of medical interventions were matched with transcripts for further analysis.

Following this first round of coding we isolated all segments of data related to vaccination and performed a more focused analysis to better understand how mothers from different social class backgrounds described their concerns and choices related to vaccination. While one question in the interview protocol asked explicitly about vaccines, we found that respondents’ discussion of vaccination was not limited to this question, and rather arose naturally in the course of conversations about making medical decisions for children.

In keeping with previous research, [34] vaccine hesitancy was defined as a spectrum of delays in acceptance or refusal of vaccines, [35] as well as those who expressed strong misgivings about the safety or efficacy of vaccines but ultimately chose to follow the recommended vaccination schedule. [24] We started by examining vaccine decisions by social class category. Four types of vaccine decisions were described by our respondents: refusal of school-mandated vaccines, refusal of seasonal influenza vaccine, delayed vaccines, and concern regarding vaccines (but no delay or refusal). We classified participants’ social class by education level and occupation. [17, 36] Middle-class mothers were defined as having completed a four-year college degree or more who were also either pursuing a conventionally white-collar profession [37] or parenting full-time. Working-class mothers were defined as those without a four-year degree who were also working in administrative or traditionally blue-collar roles [37] or parenting full time. [36] If a mother was parenting full time social class categorization was based on educational level alone.

## Results

### Participant characteristics

Interviews were conducted with 37 mothers, and lasted between one and three hours, with an average length of 81 min. Twenty-five interviews were conducted in person, with the other 12 (four middle-class respondents and eight working-class respondents) occurring over the phone due to time or distance constraints. Of the 37 participants, 22 were middle-class and 15 were working-class. The majority were white, in their thirties and forties, and had two or three children. Working-class mothers were more likely than middle-class mothers to have four or more children, reflecting higher total fertility rates among American women without a college degree.[38] Participant characteristics are summarized in Table 1.

**Table 1:** Participant Characteristics (*n* = 37)

	**Middle Class [*n* (%)]**	**Working Class [*n* (%)]**
**Race**		
White	15 (68)	12 (80)
Black	5 (23)	2 (13)
Other	2 (9)	1 (7)
Total	22 (100)	15 (100)
**No. of Children (age < 1–19 years)**		
1 child	4 (18)	0 (0)
2 children	11 (50)	4 (27)
3 children	6 (27)	2 (13)
4 + children	1 (5)	9 (60)
Total	22 (100)	15 (100)
**Occupation**		
Education	7 (32)	0 (0)
Childcare	0 (0)	1 (7)
Social work	2 (9)	0 (0)
Healthcare	2 (9)	0 (0)
Administrative work	1 (5)	2 (13)
Retail	0 (0)	1 (7)
Full-time parenting	7 (32)	10 (67)
Other	3 (14)	0 (0)
Total	22 (101)^a^	15 (100)
**Age**		
20–29	1 (5)	1 (7)
30–39	11 (50)	2 (13)
40–49	8 (36)	8 (53)
50 +	2 (9)	4 (27)
Total	22 (100)	15 (100)

^a^Total does not equal 100 due to rounding of percentages

The vaccine decisions of participants mapped to social class background are summarized in Table 2. Most participants [22] fell into multiple categories – for example, mothers who refused a school-mandated vaccine and the influenza vaccine for their child(ren).

**Table 2:** Vaccine Decisions by Social Class Category (Total *n* = 37; Middle Class *n* = 22; Working Class *n* = 15)

	**Middle-Class Participants [*n* (%)]**^**a**^	**Working-Class Participants [*n* (%)]**^**b**^
Refused School-Mandated Vaccine(s)	8 (36)^c^	10 (67)^c^
Refused Seasonal Influenza Vaccine	13 (59)^c^	13 (87)^c^
Delayed Vaccine(s)	10 (46)^c^	3 (20)^c^
Vaccine Concerned, No Delay/Refusal	3 (14)^c^	1 (7)^c^

^a^Column does not sum to 22 since a participant can be counted in multiple categories

^b^Column does not sum to 15 since a participant can be counted in multiple categories

^c^Percentages reflect the proportion of participants within each social class category

### Class, vaccination, and power in parental relationships with institutional authorities

Analysis of the variation in experiences recounted by the middle- and working-class mothers in our study demonstrated that vaccine decisions were influenced by mothers’ relationship to social institutions (e.g. schools, medical care, law enforcement). We found that when mothers described their feelings about the context in which these decisions were made, they invoked distinct levels of authority in relation to social institutions. This influenced their perceptions of the actions available to them when considering vaccination for their children.

Mothers we spoke with discussed these relationships, and the power dynamics therein, through three types of interactions: 1) interactions with pediatric clinicians; 2) interactions with school administrators; and 3) interactions with emergency room staff. These interactions highlighted the ways in which the middle- and working-class mothers in our study felt differently empowered and constrained in their vaccine choices, ultimately impacting their experiences of vaccine decision-making.

### Interactions with pediatric clinicians

Mothers we interviewed from both the middle and working classes reported clinician pushback on their vaccine hesitancy. Importantly, however, mothers’ responses to that pushback differed, specifically regarding concerns about the consequences of refusing vaccines. Middle-class mothers typically viewed these discussions with clinicians as irritating, saying that medical professionals were “shaming” them, or repeatedly pushing them to vaccinate:

*Every time we are at the pediatrician they’re like, “You all need to get flu shots, get them right now.” You know, it’s, “What? You haven’t gotten your flu shot yet? What’s wrong with you?” …I mean, the flu shot comes with a lot of shaming…We had a doctor literally shame [my husband] in the office and then she felt the need to call me afterwards because – I don’t quite know why!* [exasperated tone, rolling her eyes].

*With the pediatrician, she asked about vaccinations the first time, and I said, “No.” She has not asked again. [But] the nurses, they kind of are like, “Well, why aren’t you vaccinating? Are you going to vaccinate? What are you going to do for school?” I get it every single time. I just say, “Right now I’m not vaccinating,” and they’ll be like, “Well, what about later?” I’m like, “We’ll think about that later.”*

While frustrating, these mothers did not perceive clinicians’ feedback as fundamentally coercive. Indeed, they felt comfortable refusing vaccines, even in the context of social judgement and repeated appeals to vaccinate.

By contrast, working-class mothers perceived clinician pushback as coercive, and even threatening. Rather than the annoyance of their middle-class counterparts, these mothers felt that clinicians used authoritative tones to pressure them into vaccine compliance.

*I felt very scare-tactic’d into [vaccines]. Without so many words, “You’re going to kill your children. You’re a bad mom and you need to do this. I’m a doctor, I know better.”*

Three working-class mothers voiced concerns specifically that clinicians would act as reporters for Child Protective Services (CPS), while six others noted concerns about potential state intervention regarding parents’ medical choices. Discussing her interest in complementary and alternative medicine (CAM) for immunity instead of vaccines, one mother described her fears:

*It’s very hard to find a doctor that doesn’t look at you like you’re crazy if you bring up anything [about] natural [medicine]. When you have young children you’re even scared to ask because you don’t know if they’re going to be very watchful and call Child Protective Services on you or something like that.*

In expressing these concerns, working-class mothers in our study described a feeling of vulnerability in relation to medical and state authorities. This perceived vulnerability impacted their experience of vaccine decision-making.

### Interactions with school administrators

Securing paperwork for school vaccination exemptions was another interaction that revealed divergent and class-based relationships with institutional authorities among mothers we interviewed. Religious exemptions were the only non-medical option available in respondents’ states, and several mothers from both class backgrounds used religious beliefs – whether or not sincerely held, by their own admission – to avoid vaccination for their children. One middle-class mother described the process of seeking a religious vaccine exemption for school:

*The schools do require vaccinations, but they allow religion as an exemption. There is a place locally that you can actually get a religious exemption. It’s literally an online church. It’s $75, I think, for a lifetime membership. You just send [the school] your membership with a seal and everything. I handed it in to my one daughter’s daycare, and they didn’t ask any questions. There’s no meeting place or anything [for the church]…They have them in every state…So we’re into it. I’m not declaring it as my religion, but it works [for the schools].*

However, while middle-class mothers in our study felt entitled to use whatever means necessary to secure their desired goal, working-class mothers were more hesitant. Even when employing the same rationale for vaccine refusals as middle-class mothers, working-class mothers were uniquely worried about state interventions in their families.

*You can’t take a philosophical exemption in my state, but you can do a religious exception. That’s what I claimed because the MMR immunization, I’ve done a lot of research on it, but that’s the one that I specifically know that they have used stem cells from aborted baby tissue to manufacture that at some point in time. They may not anymore, but my point is, I’m not even going to mess with that. I also feel like, if I started immunizing my kids now, if I ever had to stand before a judge he would say, “Well, why are you immunizing them now when you couldn’t before?” So I’m trying to keep that continuity.*

This mother shared her belief that she must maintain consistency in her expressed vaccine hesitancy to avoid future censure from legal authorities, a concern not expressed by middle-class mothers. For those among our respondents who did not seek vaccine exemptions, working-class mothers were also the only to report not doing so because they viewed school vaccines as truly compulsory:

*For the kids I do [all the vaccines]. It’s a requirement. They won’t even let you in the school if your stuff is not done. [My kids] get all of [the vaccines]. I don’t particularly want them to, but I’m only doing it because I think it’s required.*

The view that vaccines could be compulsory highlights the constraint in vaccine decision-making experiences among these working-class mothers.

### Interactions with emergency room personnel

Finally, middle- and working-class mothers in our study described distinct thought processes around vaccine hesitancy in relation to potential engagement of their child with emergency medical services, in hospital-based emergency room settings, for any medical needs (not only care for vaccine-preventable illness). Middle-class mothers described approaching interactions with clinicians in emergency rooms on their own terms, seeking advice but ultimately viewing medical decisions as an individual choice.

*Our pediatrician now knows our family, and she knows how we want to do an alternative science [of medicine] and so she'll talk to us in that way…”You guys aren't [going to vaccinate], and so I'm gonna talk to you in this way.” But she wasn't putting us down in the way that a lot of doctors do. Yeah, it was like, “If you go to an emergency room, you need to tell them this because your child doesn't have the traditional vaccines that another child would and you need to tell them this is what you want to do because of that.”*

Middle-class mothers in our study expressed autonomy in their relationship to emergency medical staff, including the right to dictate care based on their vaccine choices. Importantly, these mothers did not report concerns about the emergency room as a contact point for CPS intervention based on vaccination status.

By contrast, in our interviews working-class mothers viewed the emergency room as fraught with opportunities for authorities to intervene. For example, one working-class mother described her concern when taking her partially vaccinated son to the emergency room:

*I started getting a little concerned, because I’m low income; I don’t want be looked at as negligent. That’s the worry that I have, is that [the emergency room clinicians] say I’m neglecting my children, and then the next thing you know, I’m going to get in some type of trouble…I do have a right of not taking [vaccines], but if you don’t know that, then they can come to you [and say], “Oh, it’s neglect.”*

For some of the working-class mothers we spoke with, refusing vaccines had ramifications for their perceived ability to obtain other forms of medical care, including emergency care, because they viewed medical staff as a contact point for potential state intervention. These concerns were not shared by our middle-class respondents.

## Discussion

This qualitative study compared the perspectives and experiences of vaccine-hesitant mothers from working- and middle-class backgrounds. We found that among the mothers with whom we spoke, vaccine decisions were at least partially shaped by differing perceptions of their own authority in medical decision-making vis-à-vis institutional authorities. Specifically, mothers discussed these relationships, and the power dynamics therein, through three types of interactions: 1) interactions with pediatric clinicians; 2) interactions with school administrators; and 3) interactions with emergency room staff.

Though past medical research has explored the relationship between vaccine decision-making and SES, [6, 34] the experiences and perspectives underlying that decision-making remain unclear. We argue that drawing on the notion of social class can help illuminate how and why parents of different social statuses may experience vaccine decision-making in distinct ways. Considering social class is important because it incorporates a degree of analytical complexity that is missed in discussions of SES. [15, 19, 20] Notably, while the concept of SES divides individuals along a spectrum according to factors like income and education, it does not infer conceptual significance to how or why the resulting categories are socially meaningful within themselves or in relation to one another. [19] By contrast, social class draws on a notion of shared culture to conceptualize how and why those metrics shape collective behavior and relationships to other social actors, including institutional authorities like school administrators, pediatricians and emergency medical clinicians. [17, 36].

Indeed, our findings suggest that social class may be one factor shaping the experience of vaccine hesitancy by impacting mothers’ relationships with institutional authorities that they perceive as having more or less power in their decision-making. As sociological work on the strategies of middle-class child-rearing would suggest, middle-class mothers in our study typically viewed medical and educational institutions as instruments of their larger parenting goals, regarding vaccines as one in a series of decisions geared towards individual child development. [11, 36] Middle-class mothers in our study did not worry about state interventions in their parenting, and felt empowered to make decisions counter to medical recommendations. [11] Conversely, working-class mothers with whom we spoke expressed anxiety regarding the possibility of state intervention. The perception that vaccine choices came with a degree of punitive risk – and specifically the perception that clinicians might increase that risk – was detrimental to these working class mothers’ trust in medical professionals.

Ultimately, our findings suggest that social class may influence how mothers interact with clinicians around vaccine decisions in ways that have important ramifications for the delivery of quality care. Though the majority of middle- and working-class mothers in our study ultimately refused one or more vaccines, they approached communications with clinicians in different ways, and brought distinct perspectives to those interactions that may impact their trust in clinicians going forward.

Our findings suggest that clinicians can improve trust by validating parents’ concern for their children’s wellbeing while avoiding coercive language. Fostering positive communication is vital because research shows that negative or strained communication between clinicians and patients can prove detrimental to health outcomes over time by reducing patient trust. [39] Patients who trust their physicians report higher health-related quality of life, [40] clinical setting satisfaction, [41] treatment adherence, [39] disclosure of health information, [42] and help-seeking. [43] The goal is not to make it easier for vaccine-hesitant parents to request exemptions, but rather to build mutually respectful relationships that improve parents’ trust in their clinicians’ expertise while encouraging them to vaccinate per clinical recommendations. Given past work showing that vaccine hesitancy is related to distrust of medical authorities, [24] building trust could be one of the most worthwhile strategies to improve vaccination rates among both working- and middle-class families.

Strategizing how to build trust through clinician-parent communication presents challenges. Past research suggests that presumptive, rather than participatory, language is associated with vaccine acceptance, [44] which might suggest that parent-centered discussion styles are detrimental to vaccination rates. Yet participatory language is consistently associated with higher patient ratings of the visit experience, and with greater levels of parent/patient trust. [45] This seeming paradox may foster concerns that two key outcomes of clinical interest – vaccine acceptance and parent satisfaction – are mutually exclusive. Our findings suggest this need not be the case. Indeed, this study can inform intervention design by encouraging language around vaccines that is presumptive while refraining from coercion or judgment, and that remains participatory in the more clinically value-neutral components of well-child visits like conversations about sleep and feeding. [46, 47] Our recommendations dovetail with research suggesting that presumptive language be framed within positive wording, eye contact, sitting on the same level as the patient, and allowing time for parents to respond, all of which build rapport without sacrificing vaccine uptake. [44].

The qualitative data presented here offers valuable considerations for clinicians striving to communicate with vaccine-hesitant parents from different class backgrounds. However, the study also has limitations. First, the use of a qualitative design limits our ability to generalize our findings to mothers outside of the greater Philadelphia region. Second, we focused on the experiences of mothers rather than fathers or other caretakers. It is possible that fathers or other caretakers might have different orientations to the institutional authorities we describe in this study. Future research should investigate the perspectives of caregivers from different roles. Finally, our classifications of working and middle class – defined in terms of education and occupation– may be incomplete. For example, this study included a higher proportion of mothers who parent full-time (46 percent) than do nationwide (29 percent [48]), though our data does reflect national findings that mothers parenting full-time are less likely to have completed college than mothers working outside the home part- or full-time.[48] Our higher proportion of full-time parents comes from sampling mothers involved in a Christian church community whose members support traditional gender roles. However, further research might fruitfully include mothers in a wider range of occupations, as well as data on income and Medicaid status to elucidate social class designations.

## Conclusion

In our study, vaccine-hesitant mothers from working- and middle-class backgrounds reported different experiences of vaccine decision-making within the context of their relationship to medical, educational, and state authorities. Their different experiences highlight factors that may affect vaccine choices, and have ramifications for how clinicians approach vaccine-hesitant mothers more broadly. Specifically, our findings suggest that clinicians should be sensitive to coercive language with working-class mothers. In light of concerns about state intervention among the working-class mothers we spoke with, receiving less pushback to clinical recommendations from these parents does not necessarily imply trust in providers. Indeed, clinicians should strive to maintain open communication regarding vaccines as a way to build trust with vaccine-hesitant parents and secure positive relationships – and outcomes – long term.

## Supplementary Information

**Additional file 1.** Interview guide for parent participants.

## Data Availability

The datasets generated and analyzed during this study are not publicly available due to the sensitive nature of the data and ethics restrictions on data sharing. Respondents did not consent to have their data publicly shared. A de-identified dataset may be available from the corresponding author on reasonable request.

## Abbreviations

CPS: Child Protective Services
CAM: Complementary and Alternative Medicine
HepB: Hepatitis B [Vaccine]
MMR: Measles, Mumps and Rubella [Vaccine]
SES: Socio-Economic Status
