# Supplementary material for: The influence of social class and institutional relationships on the experiences of vaccine-hesitant mothers: a qualitative study
Source: BMC Public Health. 2022 Dec 9;22:2309. doi: 10.1186/s12889-022-14420-1 (PMC9733306; doi:10.1186/s12889-022-14420-1)
Supplement: Supplementary file 1 — Additional file 1. Interview guide for parent participants. [file 12889_2022_14420_MOESM1_ESM.docx]

**INTERVIEW GUIDE FOR PARENT PARTICIPANTS**

**University of Pennsylvania**

**Project Title: Health Choices in Family Life**

1. Tell me a little bit about yourself.
   1. Where do you live now? (If interviewing at home, ask about when respondent moved here, where from, etc.)
   2. What do you do for a living?
   3. What has been your trajectory with education and employment?
      1. Where did you go to school? (High school, college, any grad school or training?)
      2. What is your highest level of education?
2. Tell me a little bit about your family growing up.
   1. How many siblings did you have (if any)?
   2. What did your parents do for a living?
   3. Was religion a part of your home growing up? (What denomination, how often attended, what role in your education?)
3. What was it like to get sick in your family when you were growing up? How did your family typically treat an illness?
   1. Did you go to the doctor?
   2. Can you give you me an example of typical time you were sick as a child and how it was treated?
   3. Were you vaccinated as a child?
4. Now I want to talk a little bit about your family today.
   1. How many children do you have? How old are they?
   2. Are you married/partnered?
   3. Are you or your family involved in a religious community these days? (If so, which denomination, how often do you attend, how important is it to you?)
5. What is it like when a family member (you, your partner, your children) gets sick?
   1. Who in your family would you say makes most of the decisions about how and when to treat an illness?
   2. Do you have a doctor or other medical practitioner whom you or your family members see regularly? Who is that doctor? How did you find him/her? How would you describe your relationship with that doctor?
6. Can you describe a recent illness in your family and how it was treated?
7. Would you say you treat illness differently for yourself compared to your children? If so, in what ways does that treatment differ?
   1. Are there interventions that you feel are okay for adults but not for children?
8. In general, how would you say you make decisions about what medicines or interventions to use?
9. Have you ever disregarded or refused a treatment that your doctor recommended for you or your kids? (If yes, please tell me more about that: How did you come to that decision?)
10. Have you ever used alternative or complementary medicine? This could include going to a chiropractor, getting acupuncture, taking herbs, visiting a massage therapist, or using any other healing strategies outside of a conventional doctor’s office.
    1. If so, how did you learn about that form of alternative medicine?
    2. How would you describe your experience with alternative medicine?
    3. Do you think your experience with alternative medicine change will change the types of medical help you seek in the future?
11. Do you think your view of health has changed over time? If so, how/why?
    1. Are there any specific events that you would describe as very important to your perspective on health, or would you describe your perspective as evolving gradually?
12. [If applicable] What has/have your experiences with childbirth been like? Did those experiences change your perspective on healthcare in any way?
13. Is there anything else that you think I should know about your experience with health and healthcare?
14. Do you have any questions for me that I can answer for you?

Thank you so much for your participation in the study! Please feel free to contact me any time using the information on the sheet that I gave you if you have anything you think of that you’d like to share, or if you have any questions that I can answer for you. I sincerely appreciate your time and help.
